# Single-step genomic evaluation with metafounders for feed conversion ratio and average daily gain in Danish Landrace and Yorkshire pigs

**DOI:** 10.1186/s12711-021-00670-x

**Published:** 2021-10-07

**Authors:** Chuanke Fu, Tage Ostersen, Ole F. Christensen, Tao Xiang

**Affiliations:** 1 Key Laboratory of Agricultural Animal Genetics, Breeding and Reproduction of Ministry of Education & Key Laboratory of Swine Genetics and Breeding of Ministry of Agriculture, Huazhong Agricultural University, Wuhan, 430070 China; 2 SEGES, Danish Agriculture & Food Council F.m.b.A., Agro Food Park 15, 8200 Aarhus N, Denmark; 3 Center for Quantitative Genetics and Genomics, Aarhus University, Blichers Alle 20, 8830 Tjele, Denmark

## Abstract

**Background:**

The single-step genomic best linear unbiased prediction (SSGBLUP) method is a popular approach for genetic evaluation with high-density genotype data. To solve the problem that pedigree and genomic relationship matrices refer to different base populations, a single-step genomic method with metafounders (MF-SSGBLUP) was put forward. The aim of this study was to compare the predictive ability and bias of genomic evaluations obtained with MF-SSGBLUP and standard SSGBLUP. We examined feed conversion ratio (FCR) and average daily gain (ADG) in DanBred Landrace (LL) and Yorkshire (YY) pigs using both univariate and bivariate models, as well as the optimal weighting factors (ω), which represent the proportions of the genetic variance not captured by markers, for ADG and FCR in SSGBLUP and MF-SSGBLUP.

**Results:**

In general, SSGBLUP and MF-SSGBLUP showed similar predictive abilities and bias of genomic estimated breeding values (GEBV). In the LL population, the predictive ability for ADG reached 0.36 using uni- or bi-variate SSGBLUP or MF-SSGBLUP, while the predictive ability for FCR was highest (0.20) for the bivariate model using MF-SSGBLUP, but differences between analyses were very small. In the YY population, predictive ability for ADG was similar for the four analyses (up to 0.35), while the predictive ability for FCR was highest (0.36) for the uni- and bi-variate MF-SSGBLUP analyses. SSGBLUP and MF-SSGBLUP exhibited nearly the same bias. In general, the bivariate models had lower bias than the univariate models. In the LL population, the optimal ω for ADG was ~ 0.2 in the univariate or bivariate models using SSGBLUP or MF-SSGBLUP, and the optimal ω for FCR was 0.70 and 0.55 for SSGBLUP and MF-SSGBLUP, respectively. In the YY population, the optimal ω ranged from 0.25 to 0. 35 for ADG across the four analyses and from 0.10 to 0.30 for FCR.

**Conclusions:**

Our results indicate that MF-SSGBLUP performed slightly better than SSGBLUP for genomic evaluation. There was little difference in the optimal weighting factors (ω) between SSGBLUP and MF-SSGBLUP. Overall, the bivariate model using MF-SSGBLUP is recommended for single-step genomic evaluation of ADG and FCR in DanBred Landrace and Yorkshire pigs.

## Background

Single-step genomic best linear unbiased prediction (SSGBLUP), as a standard genomic evaluation method using single nucleotide polymorphism (SNP) genotypes, has been successfully used in the pig industry [1]. By using SSGBLUP, genomic selection (GS) can be implemented even if some animals are not genotyped because it can integrate phenotypic records, pedigree, and genomic information on all relevant animals [2, 3]. However, some problems with SSGBLUP need to be solved. First, theoretically, the allele frequencies used to construct the genomic relationship matrix should be those of the base population of the pedigree [4]. However, allele frequencies in the base populations are usually unknown, because animals in the base population are often not genotyped [5]. Using allele frequencies other than those of the base population for the construction of the genomic relationship matrix causes incompatibility between the pedigree-based and marker-based relationship matrices [6]. Second, to make the genomic relationship matrix invertible and capture genetic variance that cannot be captured by SNPs, the genomic relationship matrix is usually combined with the pedigree-based relationship matrix, using a weighting factor (ω) that may be breed- and trait-specific, which needs to be further investigated. Some approaches are available to address these two problems, but they are not perfect. For example, instead of allele frequencies in the base population, those of the genotyped population are used to construct the genomic relationship matrix. Other studies have adjusted the marker-based relationship matrix to make it compatible with the pedigree-based relationship matrix [7–9]. Some studies use the same weighting factor (ω) for different traits, leading to a decrease in the accuracy of estimated breeding values [10]. Although these solutions have been widely used in SSGBLUP and appear to be effective in practice, they do not fully solve the above-mentioned issues and further developments are needed to improve SSGBLUP.

To address the issue of unknown base allele frequencies and incompatibility of genomic and pedigree relationship matrices, Christensen [6] and Legarra et al. [5] defined a metafounder as a finite-sized pool of gametes from which alleles are randomly extracted to form diploids that represent animals in the base population [5]. Based on metafounder theory [5, 6], allele frequencies of 0.5 should be used to construct the genomic relationship matrix and referred to in the construction of the pedigree-based relationship matrix, such that compatibility between pedigree-based and marker-based relationship matrices is automatically achieved. Thus, metafounder theory solves the two above-mentioned issues of SSGBLUP.

Since this theory was formulated, a few studies have focused on the application of single-step genomic evaluation coupled with metafounders, i.e. MF-SSGBLUP. For instance, Garcia-Baccino et al. [11] used simulated data to investigate methods to estimate relatedness in the base population (γ). Using MF-SSGBLUP, Xiang et al. [12] estimated breeding values for total number of piglets born in purebred pigs for two-way crossbred performance, and van Grevenhof et al. [13] estimated breeding values of purebred pigs for three-way crossbred performance, based on simulated data. In dairy cattle, Bradford et al. [14] compared the predictive performance of SSGBLUP with unknown parent groups and MF-SSGBLUP using simulated data. Kudinov et al. [15] recommended MF-SSGBLUP for genomic prediction in red dairy cattle. Macedo et al. [16] reported that MF-SSGBLUP can yield unbiased estimates of breeding values when pedigree information is not complete.

Studies that compare MF-SSGBLUP and standard SSGBLUP for genomic evaluation on production traits such as feed conversion rate (FCR) and average daily gain (ADG) are lacking in pigs. In addition, only a few studies have applied these two methods in a multi-trait model, which is extensively used in pig breeding programs. Finally, the effect of using different weighting factors ω for the pedigree-based relationship matrix in MF-SSGBLUP on genomic predictions has not been investigated. Therefore, the aims of this study were to (1) compare estimates of genetic parameters for ADG and FCR obtained with SSGBLUP and MF-SSGBLUP; (2) investigate and compare the accuracy and bias of GEBV using SSGBLUP and MF-SSGBLUP with univariate and bivariate animal models; and (3) find optimal weighting factors, ω, for ADG and FCR when using SSGBLUP or MF-SSGBLUP.

## Methods

### Phenotypic records

All datasets were provided by the SEGES Danish Pig Research Centre. The traits ADG (ADG = weight gain/days) and FCR (FCR = feed intake/weight gain) were measured during the 30 to 100 kg interval for both DanBred Landrace (LL) and Yorkshire (YY) pigs between the years 2000 and 2017. Records for ADG were available on 686,420 LL pigs, of which 18,889 also had FCR records, and on 570,493 YY pigs, of which 19,387 also had FCR records. Pedigrees of the two breeds were traced back to January 1, 1994, and included 700,960 LL pigs and 582,114 YY pigs.

### Genotypes

In total, 37,699 LL and 37,845 YY pigs were genotyped with the Illumina PorcineSNP60 Genotyping BeadChip, with approximately equal numbers by breed using version 1 and version 2. SNPs were mapped to chromosomes based on the pig genome build 10.2 [17]. Genotype quality control was conducted separately for the two versions and the two breeds. First, animals with a genotype call rate lower than 90% were excluded. Then, SNPs with a genotype call rate lower than 90% and a minor allele frequency lower than 0.05 were filtered out, as were SNPs that deviated strongly from Hardy Weinberg equilibrium within breed (p < 10e−7). Fimpute v2.2 [18] was used to impute missing genotypes. After quality control, 37,621 SNPs for 37,699 LL pigs and 36,687 SNPs for 37,845 YY pigs were retained for further analysis.

### Methods to construct the relationship matrix

For SSGBLUP, the inverse of the combined genomic and pedigree-based relationship matrix (${\mathbf{H}}^{ - 1}$) was constructed following Christensen and Lund [3] and Christensen et al. [9], as:

$${\mathbf{H}}^{ - 1} = \left[ {\begin{array}{*{20}c} {\mathbf{0}} & {\mathbf{0}} \\ {\mathbf{0}} & {{\mathbf{G}}_{{{\text{a}},\upomega }}^{ - 1} - {\mathbf{A}}_{22}^{ - 1} } \\ \end{array} } \right] + {\mathbf{A}}^{ - 1} ,$$

where ${\mathbf{G}}_{{{\text{a}},\upomega }} = \left( {1 -\upomega } \right){\mathbf{G}}_{{\text{a}}} +\upomega {\mathbf{A}}_{22}$ and ${\mathbf{A}}_{22}$ contains the pedigree-based relationships among the genotyped pigs. Matrix ${\mathbf{G}}_{{\mathbf{a}}}$ is an adjusted version of the genomic relationship matrix ${\mathbf{G}}$, that was constructed following VanRaden’s [4] method 1 as: ${\mathbf{G}} = \frac{{{\mathbf{ZZ^{\prime}}}}}{{2\sum p_{i} \left( {1 - p_{i} } \right)}}$, where ${\mathbf{Z}}$ is a matrix with entries 0–2$p_{i}$, 1–2$p_{i}$, 2–2$p_{i}$ for genotypes *AA*, *AB*, and *BB*, respectively, for SNP $i$ ranging from 1 to $m$, where $m$ is the number of markers. Theoretically, $p_{i}$ are the minor allele frequencies in the base population but, in practice, $p_{i}$ is often computed from the observed genotypes, and this was the case in our study. To be compatible with the pedigree-based relationship matrix, matrix ${\mathbf{G}}$ was scaled to create a matrix ${\mathbf{G}}_{{\text{a}}}$, following the method described by Christensen et al. [9], as: ${\mathbf{G}}_{{\text{a}}} =\upbeta {\mathbf{G}} + {\upalpha }{\mathbf{11^{\prime}}}$, where β and ${\upalpha }$ are obtained by solving the following equations: $Avg\left( {diag\left( {\mathbf{G}} \right)} \right)\upbeta + {\upalpha } = Avg\left( {diag\left( {{\mathbf{A}}_{22} } \right)} \right)$ and $Avg\left( {\mathbf{G}} \right)\upbeta + {\upalpha } = Avg\left( {{\mathbf{A}}_{22} } \right)$. The inverse of the pedigree-based relationship ${\mathbf{A}}$ matrix was constructed according to Henderson’s rule [19] by considering inbreeding coefficients in the relationship matrix computation. Matrix ${\mathbf{A}}_{22}$, with inverse ${\mathbf{A}}_{22}^{ - 1}$, was constructed following Colleau [20]. To investigate the effects of different weighting factors, ω, between the pedigree-based and genomic relationship matrices on genomic predictions in SSGBLUP, we tested values of ω ranging from 0.05 to 0.95 with an interval of 0.05.

For MF-SSGBLUP, parameter γ, which represents the relatedness of the animals in the LL and YY base populations, must be estimated to construct the inverse of the combined relationship matrix. Christensen [6] and Garcia-Baccino et al. [11] reported that $\upgamma = 8\sigma_{p}^{2}$ where $\sigma_{p}^{2}$ is the variance of the true allele frequencies in the base population. To estimate γ, $\sigma_{p}^{2}$ was calculated by the method of generalized least squares reported by Garcia-Baccino et al. [11] and Xiang et al. [12]. For this purpose, gene content at a SNP, i.e. the number of copies of a given reference allele (e.g. 0/1/2 for genotypes *AA*/*AB*/*BB*) was viewed as a quantitative trait with a heritability of 1, and all the variance was assumed to be additive genetic [21]. The linear model used for analysis of gene content, which was independently proposed by McPeek et al. [22] and Gengler et al. [23], was:

$${\mathbf{m}}_{i} = {\mathbf{1}}\mu_{i} + {\mathbf{Wu}}_{i} + {\mathbf{e}}_{i} ,$$

where ${\mathbf{m}}_{i}$ is a vector of gene contents for SNP $i$ across all animals; $\mu_{i}$ is the overall mean of gene content, with $\mu_{i} = 2p_{i}$, where $p_{i}$ is the allele frequency in the base population; ${\mathbf{u}}_{i}$ is the vector of random genetic effects and was assumed to follow a multivariate normal distribution ${\mathbf{u}}_{i} \sim N\left( {{\mathbf{0}},{\mathbf{A}}\sigma_{u}^{2} } \right)$ [24], where ${\mathbf{A}}$ is the pedigree-based additive genetic relationship matrix; matrix ${\mathbf{W}}$ is an incidence matrix relating individuals to their genotypes; and ${\mathbf{e}}_{i}$ is a vector of error terms. We used $\sigma_{u}^{2} = 0.999$ and $\sigma_{e}^{2} = 0.001$, such that heritability was almost 1. Similar to Xiang et al. [25], the overall mean $\mu_{i}$ for each locus $i$ was estimated as the best linear unbiased estimate (BLUE) using the BLUPF90 [26] software. Then, the estimate of the base allele frequency ${ }\hat{p}_{i}$ was calculated as half of the $\hat{\mu }_{i}$. The variance of base population allele frequencies, $\widehat{{\sigma_{p}^{2} }}$, was obtained, followed by $\widehat{\gamma } = 8\widehat{{\sigma_{p}^{2} }}$.

After estimating γ, the inverse combined relationship matrix ${\mathbf{H}}(\upgamma )^{ - 1}$ was constructed for the LL and YY populations as follows:

$${\mathbf{H}}\left( {\widehat{\upgamma }} \right)^{ - 1} = \left[ {\begin{array}{*{20}c} {\mathbf{0}} & {\mathbf{0}} \\ {\mathbf{0}} & {{\mathbf{G}}_{{{\text{MF}},\upomega }}^{ - 1} - {\mathbf{A}}\left( {\widehat{\upgamma }} \right)_{22}^{ - 1} } \\ \end{array} } \right] + {\mathbf{A}}\left( {\widehat{\upgamma }} \right)^{ - 1} ,$$

where ${\mathbf{G}}_{{{\text{MF}},\upomega }} = \left( {1 -\upomega } \right){\mathbf{G}}_{{{\text{MF}}}} +\upomega {\mathbf{A}}\left( {\widehat{\gamma }} \right)_{22}$, and ${\mathbf{A}}\left( {\widehat{\upgamma }} \right)_{22}$ contain the pedigree-based relationships among the genotyped pigs. The marker-based relationship matrix was derived as ${\mathbf{G}}_{\text{MF}} = {{\left( {{\mathbf{M}} - {\mathbf{1}}_{m} {\mathbf{1^{\prime}}}_{n} } \right)\left( {{\mathbf{M}} - {\mathbf{1}}_{m} {\mathbf{1^{\prime}}}_{n}} \right)^{\prime } } \mathord{\left/ {\vphantom {{\left( {{\mathbf{M}} - {\mathbf{1}}_{m} {\mathbf{1^{\prime}}}_{n} } \right)\left( {{\mathbf{M}} - {\mathbf{1}}_{m} {\mathbf{1^{\prime}}}_{n}} \right)^{\prime } } s}} \right. \kern-\nulldelimiterspace} s}$, where ${\mathbf{M}}$ is a matrix with entries 0, 1, and 2 for genotypes *AA*, *AB*, and *BB*, respectively; ${\mathbf{1}}_{m}$ and ${\mathbf{1}}_{n}$ are vectors of ones of lengths $m$ for the number of markers and $n$ for the number of animals, respectively; and $s$ is a scaling parameter equal to $\frac{m}{2}$ [27]. The calculation of ${\mathbf{H}}\left( {\widehat{\upgamma }} \right)^{ - 1}$ was based on the method reported by Garcia-Baccino et al. [11]. Values of ω ranging from 0.05 to 0.95 with an interval of 0.05 were investigated. The inverse of the pedigree-based relationship matrix ${\mathbf{A}}\left( {\widehat{\upgamma }} \right)^{ - 1}$ was constructed according to Henderson’s [19] rules and matrix ${\mathbf{A}}\left( {\widehat{\upgamma }} \right)_{22}$ was constructed following the method described by Colleau [20], with a minor modification based on metafounders [5]. Matrix ${\mathbf{A}}\left( {\widehat{\upgamma }} \right)_{22}^{ - 1}$ is the inverse of ${\mathbf{A}}\left( {\widehat{\upgamma }} \right)_{22}$.

### Statistical models

Both univariate and bivariate animal models were applied to estimate breeding values for ADG and FCR for both LL and YY pigs. The statistical models for analysis of ADG and FCR were as follows:

$${\mathbf{y}}_{ADG} = {\mathbf{X}}_{ADG}{\mathbf{\upbeta}}_{ADG} + {\mathbf{Z}}_{ADG} {\mathbf{a}}_{ADG} + {\mathbf{K}}_{ADG} {\mathbf{l}}_{ADG} + {\mathbf{W}}_{ADG} {\mathbf{p}}_{ADG} + {\mathbf{e}}_{ADG} ,$$

$${\mathbf{y}}_{FCR} = {\mathbf{X}}_{FCR}{\mathbf{\upbeta}}_{FCR} + {\mathbf{Z}}_{FCR} {\mathbf{a}}_{FCR} + {\mathbf{e}}_{FCR} ,$$

where ${\mathbf{y}}_{ADG}$ and ${\mathbf{y}}_{FCR}$ are vectors of phenotypic records for ADG and FCR, respectively; ${\mathbf{\upbeta}} _{ADG}$ and ${\mathbf{\upbeta}} _{FCR}$ are the vectors of fixed effects, which include contemporary groups effects (animals present at the same point of time), sex effect, and covariates on initial weights for ADG and FCR, respectively; ${\mathbf{a}}_{ADG}$, ${\mathbf{a}}_{FCR}$, ${\mathbf{l}}_{ADG}$, and ${\mathbf{p}}_{ADG}$ are the vectors of random effects, with ${\mathbf{a}}_{ADG}$ and ${\mathbf{a}}_{FCR}$ representing the additive genetic effects for ADG and FCR, respectively; ${\mathbf{l}}_{ADG}$ and ${\mathbf{p}}_{ADG}$ are vectors of random birth litter effects and random pen effects for ADG; ${\mathbf{X}}$, ${\mathbf{Z}}$, ${\mathbf{K}}$, ${\mathbf{W}}$ are the corresponding incidence matrices; and ${\mathbf{e}}_{ADG}$ and ${\mathbf{e}}_{FCR}$ are vectors of random residual effects. Random litter and pen effects were assumed to follow normal distributions, i.e., ${\mathbf{l}}_{ADG} \sim N\left( {{\mathbf{0}},{\mathbf{I}}\sigma_{litter}^{2} } \right)$ and ${\mathbf{p}}_{ADG} \sim N\left( {{\mathbf{0}},{\mathbf{I}}\sigma_{pen}^{2} } \right)$, where ${\mathbf{I}}$ is an identity matrix. In the univariate models, vectors ${\mathbf{a}}_{ADG}$ and ${\mathbf{a}}_{FCR}$ were assumed to be normally distributed with a mean of **0** and (co)variance structures $var\left( {{\mathbf{a}}_{ADG} } \right) = {\mathbf{H}}\sigma_{a\_UNI\_ADG}^{2}$ and $var\left( {{\mathbf{a}}_{FCR} } \right) = {\mathbf{H}}\sigma_{a\_UNI\_FCR}^{2}$ for SSGBLUP, and $var\left( {{\mathbf{a}}_{ADG} } \right) = {\mathbf{H}}(\upgamma )\sigma_{a\_UNI\_MF\_ADG}^{2}$ and $var\left( {{\mathbf{a}}_{FCR} } \right) = {\mathbf{H}}(\upgamma )\sigma_{a\_UNI\_MF\_FCR}^{2}$ for MF-SSGBLUP, where $\sigma_{a\_UNI\_ADG}^{2}$, $\sigma_{a\_UNI\_FCR}^{2}$, $\sigma_{a\_UNI\_MF\_ADG}^{2}$, and $\sigma_{a\_UNI\_MF\_FCR}^{2}$ are, respectively, the additive genetic variances for ADG and FCR in the univariate models using SSGBLUP and MF-SSGBLUP. In the bivariate model, ${\mathbf{a}}_{ADG}$ and ${\mathbf{a}}_{FCR}$ were assumed to follow a multivariate normal distribution:

$$\left( {\begin{array}{*{20}c} {{\mathbf{a}}_{ADG} } \\ {{\mathbf{a}}_{FCR} } \\ \end{array} } \right)\sim N\left( {\mathbf{0},\left( {\begin{array}{*{20}c} {\sigma_{a\_BI\_ADG}^{2} } & {\sigma_{a\_BI\_ADG,a\_BI\_FCR} } \\ {\sigma_{a\_BI\_FCR,a\_BI\_ADG} } & {\sigma_{a\_BI\_FCR}^{2} } \\ \end{array} } \right) \otimes {\mathbf{H}}} \right)\;{\text{for}\; \text{SSGBLUP}}$$

$${\text{and}}\;\left( {\begin{array}{*{20}ll} {{\mathbf{a}}_{ADG} } \\ {{\mathbf{a}}_{FCR} } \\ \end{array} } \right)\sim N\left( {\mathbf{0},\left( {\begin{array}{*{20}c} {\sigma_{a\_BI\_MF\_ADG}^{2} } & {\sigma_{a\_BI\_MF\_ADG,a\_BI\_MF\_FCR} } \\ {\sigma_{a\_BI\_MF\_FCR,a\_BI\_MF\_ADG} } & {\sigma_{a\_BI\_MF\_FCR}^{2} } \\ \end{array} } \right) \otimes {\mathbf{H}}(\upgamma )} \right)\;{\text{for}}\; {\text{MF-SSGBLUP}},$$

where $\sigma_{a\_BI\_ADG}^{2}$, $\sigma_{a\_BI\_FCR}^{2}$, $\sigma_{a\_BI\_MF\_ADG}^{2}$ and $\sigma_{a\_BI\_MF\_FCR}^{2}$ are the additive genetic variances for ADG and FCR in the bivariate model for SSGBLUP and MF-SSGBLUP, respectively, and $\sigma_{a\_BI\_ADG,a\_BI\_FCR}$ and $\sigma_{a\_BI\_MF\_ADG,a\_BI\_MF\_FCR}$ are the additive genetic covariances between ADG and FCR in the bivariate model for SSGBLUP and MF-SSGBLUP, respectively. It should be noted that the (co)variance components in MF-SSGBLUP cannot be directly compared with those in SSGBLUP based on the assumption that animals in the base population were unrelated. In MF-SSGBLUP, all the genetic (co)variance components, including the additive genetic variances for ADG and FCR and the genetic covariance between ADG and FCR, were multiplied by $\left( {1 -\upgamma /2} \right)$, with γ estimated separately for the LL and YY populations [5, 26].

### Methods and genetic parameters

Using the HE regression algorithm in the HIBLUP software (https://hiblup.github.io/), all available data for the LL and YY pigs were used to estimate (co)variance components and heritabilities ($h^{2}$) with ω = 0.25 for both ADG and FCR using four methods: (1) SSGBLUP method with a univariate model (*UNI_SSGBLUP*); (2) MF-SSGBLUP method with a univariate model (*UNI_MF-SSGBLUP*); (3) SSGBLUP method with a bivariate model (*BI_SSGBLUP*), and (4) MF-SSGBLUP method with a bivariate model (*BI_MF-SSGBLUP*). Estimation of the genetic correlation between ADG and FCR was performed only with the two bivariate methods, i.e., *BI_SSGBLUP* and *BI_MF-SSGBLUP.* The four methods were evaluated for both the LL and YY populations. For *UNI_SSGBLUP*, heritability was calculated as $h_{UNI\_ADG}^{2} = \frac{{\sigma_{a\_UNI\_ADG}^{2}\;\;\; }}{{\;\sigma_{a\_UNI\_ADG}^{2}~~~~~~~~ + \sigma_{UNI\_litter}^{2} ~~~~~~+ \sigma_{UNI\_pen}^{2}~~~~~+ \sigma_{e\_UNI\_ADG}^{2}\;\;\;\;\;\;\; }}$ and $h_{UNI\_FCR}^{2} = \frac{{\sigma_{a\_UNI\_FCR}^{2}\;\;\;\; }}{{\;\sigma_{a\_UNI\_FCR}^{2}~~~~~~~ + \sigma_{e\_UNI\_FCR}^{2} \;\;\;\;\;\;}}$ for ADG and FCR, respectively. For *BI_SSGBLUP*, heritability was calculated as $h_{BI\_ADG}^{2} = \frac{{\sigma_{a\_BI\_ADG}^{2}\;\;\;\; }}{{\;\sigma_{a\_BI\_ADG}^{2}\;\;\;\; + \sigma_{BI\_litter}^{2} \;\;\;+ \sigma_{BI\_pen}^{2} \;\;+ \sigma_{e\_BI\_ADG}^{2}\;\;\;\;\;\; }}$ and $h_{BI\_FCR}^{2} = \frac{{\sigma_{a\_BI\_FCR}^{2} \;\;\;}}{{\sigma_{a\_BI\_FCR}^{2}\;\;\;\; + \sigma_{e\_BI\_FCR}^{2}\;\;\;\;\; }}$ for ADG and FCR, respectively. For *UNI_MF-SSGBLUP* and *BI_MF-SSGBLUP*, all variance components had to be scaled, as mentioned above. Thus, for *UNI_MF-SSGBLUP*, heritability was calculated as $h_{UNI\_MF\_ADG}^{2} = \frac{{\sigma_{a\_UNI\_MF\_ADG}^{2} \;\;\;\;\;\; *\left( {1 -\upgamma /2} \right)}}{{\sigma_{a\_UNI\_MF\_ADG}^{2} \;\;\;\;\;\;*\left( {1 -\upgamma /2} \right) + \sigma_{UNI\_MF\_litter}^{2}\;\;\;\;\; + \sigma_{UNI\_MF\_pen}^{2}\;\;\;\; + \sigma_{e\_UNI\_MF\_ADG}^{2}\;\;\;\;\;\;\;\;\; }}$ and $h_{UNI\_MF\_FCR}^{2} = \frac{{\sigma_{a\_UNI\_MF\_FCR}^{2}\;\;\;\;\;\; *\left( {1 -\upgamma /2} \right)\;}}{{\;\sigma_{a\_UNI\_MF\_FCR}^{2}\;\;\;\;\;\; *\left( {1 -\upgamma /2} \right) + \sigma_{e\_UNI\_MF\_FCR}^{2}\;\;\;\;\;\;\;\; }}$ for ADG and FCR, respectively. For *BI_MF-SSGBLUP*, heritability was calculated as $h_{BI\_MF\_ADG}^{2} = \frac{{\sigma_{a\_BI\_MF\_ADG}^{2}\;\;\;\;\;\; *\left( {1 -\upgamma /2} \right)}}{{\sigma_{a\_BI\_MF\_ADG}^{2}\;\;\;\;\;\; *\left( {1 -\upgamma /2} \right) + \sigma_{BI\_MF\_litter}^{2} \;\;\;\;\;+ \sigma_{BI\_MF\_pen}^{2}\;\;\;\; + \sigma_{e\_BI\_MF\_ADG}^{2}\;\;\;\;\;\;\;\;\; }}$ and $h_{BI\_MF\_FCR}^{2} = \frac{{\sigma_{a\_BI\_MF\_FCR}^{2}\;\;\;\;\;\; *\left( {1 -\upgamma /2} \right)}}{{\sigma_{a\_BI\_MF\_FCR}^{2}\;\;\;\;\;\; *\left( {1 -\upgamma /2} \right) + \sigma_{e\_BI\_MF\_FCR}^{2} \;\;\;\;\;\;\;}}$ for ADG and FCR, respectively. The genetic correlation between ADG and FCR was calculated as $r_{g} = \frac{{\sigma_{a\_BI\_ADG,a\_BI\_FCR}\;\;\;\;\;\;\;\;}}{{\sqrt {\sigma_{a\_BI\_ADG}^{2}\;\;\;\; *\sigma_{a\_BI\_FCR}^{2}\;\;\;\;\;\; }\; }}$ and $\frac{{\sigma_{a\_BI\_MF\_ADG,a\_BI\_MF\_FCR}\;\;\;\;\;\;\;\;\;\;\; }}{{\sqrt {\sigma_{a\_BI\_MF\_ADG}^{2} \;\;\;\;\;\;*\sigma_{a\_BI\_MF\_FCR}^{2}\;\;\;\;\;\;\; }\; }}$ for *BI_SSGBLUP* and *BI_MF-SSGBLUP*, respectively.

### Predictive ability

Genomic predictions for the four methods of analysis were obtained using the preconditioned conjugate gradient algorithm in the DMU software [28], using different ω (ranging from 0.05 to 0.95). Notably, in the univariate and bivariate models, each time only one ω was used to construct the relationship matrix. Predictive abilities were determined as the correlations between corrected phenotypes (${\mathbf{Y}}_{c}$) and genomic estimated breeding values (${\hat{\mathbf{a}}}$) in different methods ($cor\left( {{\mathbf{Y}}_{{\text{c}}} ,{\hat{\mathbf{a}}}} \right)$), in a validation population, following the method reported by Christensen et al. [9]. Corrected phenotypes were obtained by adjusting for all the fixed and random effects except the additive genetic effect. To avoid biases in the calculation of ${\mathbf{Y}}_{c}$ towards a specific single-step method, a univariate pedigree-based BLUP was used to estimate all the fixed effects and random effects to obtain ${\mathbf{Y}}_{c}$. As an indicator of the dispersion bias of the genomic predictions, regression coefficients of the corrected phenotypes (${\mathbf{Y}}_{c}$) on the genomic estimated breeding values (${\hat{\mathbf{a}}}$) were calculated for each method.

Animals with phenotypes were divided into training populations and validation populations by using a cutoff date on date of birth (May 1st, 2016). The two training populations included 654,908 (29,825 genotyped) LL pigs and 541,301 (30,144 genotyped) YY pigs. The two validation populations included all pigs born between May 1st, 2016 and February 1st, 2017, i.e. 31,515 (7700 genotyped) LL pigs and 29,192 (7645 genotyped) YY pigs. Each validation population was divided into a genotyped subgroup and a non-genotyped subgroup. The numbers of animals in each dataset are summarized in Table 1. The predictive ability was investigated for each subgroup and for the total validation population for both the YY and LL populations.

**Table 1:** Numbers of animals with records for average daily gain (ADG) and feed conversion ratio (FCR) and with genotypes in the training and validation datasets for the two populations

	Training	Validation
Landrace		
ADG	654,908	31,512
FCR	17,901	988
Genotyped	29,825	7700
Genotyped and FCR	2660	902
Yorkshire		
ADG	541,301	29,192
FCR	18,478	909
Genotyped	30,144	7,645
Genotyped and FCR	2802	870

All genotyped animals have ADG records, but only some of them have FCR records

ADG average daily gain, FCR feed conversion ratio

Genotyped: genotyped individuals in the population

Genotyped and FCR: genotyped individuals with FCR records in the population

A Hotelling–Williams t-test at a 5% confidence level was performed to evaluate the statistically significant differences in predictive ability between methods. The value of ω that resulted in the highest accuracy of genomic predictions was considered the optimal ω. However, if several values of ω resulted in accuracies that were close to the highest accuracy, the value of ω with the lowest bias was considered the optimal weighting factor.

## Results

### Genetic parameters

Tables 2 and 3 show the estimated genetic parameters with a ω of 0.25 for ADG and FCR for the four methods of analysis and estimates of the genetic correlation between ADG and FCR for the bivariate analyses (*BI_SSGBLUP* and *BI_MF-SSGBLUP*) for the LL and YY populations, respectively. For MF-SSGBLUP, parameter γ was estimated at ${\hat{\gamma }}_{L}$ = 0.605 and ${\hat{\gamma }}_{Y}$ = 0.553 for the LL and YY populations, respectively.

**Table 2:** Estimates of variance components, heritabilities, and genetic correlations for average daily gain (ADG) and feed conversion ratio (FCR) in Landrace pigs using pedigree-based univariate BLUP and univariate (UNI) or bivariate (BI) single-step genomic prediction models with or without metafounders (MF)

	$\sigma_{a\_ADG}^{2}$	$\sigma_{a\_ADG, a\_FCR}$	$\sigma_{a\_FCR}^{2}$	$\sigma_{pen}^{2}$	$\sigma_{litter}^{2}$	$\sigma_{e\_ADG}^{2}$	$\sigma_{e\_FCR}^{2}$	$h_{a\_ADG}^{2}$	$h_{a\_FCR}^{2}$	$r_{g}$
Pedigree-based univariate BLUP	1916.33 (98.71)	− 0.56 (0.44)	4.12 × 10^–3^ (2.55 × 10^–3^)	633.35 (69.57)	455.57 (77.25)	4544.12 (96.21)	2.82 × 10^–2^ (2.49 × 10^–3^)	0.25 (0.05)	0.13 (0.06)	− 0.20 (0.11)
UNI_SSGBLUP	1899.91 (99.30)	–	3.44 × 10^–3^ (2.61 × 10^–3^)	669.29 (64.02)	510.24 (81.99)	4787.67 (95.34)	2.87 × 10^–2^ (2.53 × 10^–3^)	0.24 (0.04)	0.11 (0.06)	–
BI_SSGBLUP	1882.02 (96.75)	− 0.48 (0.42)	3.35 × 10^–3^ (2.42 × 10^–3^)	691.50 (50.15)	527.58 (63.81)	4885.49 (90.25)	2.88 × 10^–2^ (2.47 × 10^–3^)	0.24 (0.04)	0.10 (0.06)	− 0.19 (0.09)
UNI_MF-SSGBLUP	1885.58 (102.48)	–	3.46 × 10^–3^ (2.59 × 10^–3^)	721.22 (59.25)	529.21 (65.23)	4894.98 (95.54)	2.88 × 10^–2^ (2.61 × 10^–4^)	0.23 (0.04)	0.11 (0.07)	–
BI_MF-SSGBLUP	1887.71 (97.14)	− 0.68 (0.43)	3.48 × 10^–3^ (2.41 × 10^–3^)	727.64 (53.62)	532.84 (63.94)	4916.36 (91.40)	2.87 × 10^–2^ (2.50 × 10^–3^)	0.23 (0.05)	0.11 (0.05)	− 0.27 (0.10)

Variance components for genetic parameters correspond to the usual genetic variance (in MF-SSGBLUP, all the variance components are multiplied by ($1 - \gamma_{L} )/2$), which is the variance among unrelated individuals in the base population

$\sigma_{a\_ADG}^{2}$ is the additive genetic variance for ADG, $\sigma_{a\_FCR}^{2}$ is the additive genetic variance for FCR; $\sigma_{{a\_ADG,{ }a\_FCR}}$ is genetic covariance between ADG and FCR; $\sigma_{litter}^{2}$ is the variance of litter effect for ADG; $\sigma_{e\_ADG}^{2}$ is the residual variance for ADG; $\sigma_{e\_FCR}^{2}$ is the residual variance for FCR

Numbers between brackets are the standard errors of the corresponding parameters

**Table 3:** Estimates of variance components, heritabilities, and genetic correlations for average daily gain (ADG) and feed conversion ratio (FCR) in Yorkshire pigs using pedigree-based univariate BLUP and univariate (UNI) or bivariate (BI) single-step genomic prediction models with or without metafounders (MF)

	$\sigma_{a\_ADG}^{2}$	$\sigma_{a\_ADG, a\_FCR}$	$\sigma_{a\_FCR}^{2}$	$\sigma_{pen}^{2}$	$\sigma_{litter}^{2}$	$\sigma_{e\_ADG}^{2}$	$\sigma_{e\_FCR}^{2}$	$h_{a\_ADG}^{2}$	$h_{a\_FCR}^{2}$	$r_{g}$
Univariate animal model BLUP	2039.74 (117.88)	− 1.19 (0.22)	3.60 × 10^–3^ (6.47 × 10^–4^)	577.24 (57.33)	445.98 (62.07)	5144.01 (94.21)	1.55 × 10^–2^ (6.19 × 10^–3^)	0.25 (0.05)	0.19 (0.05)	− 0.44 (0.15)
UNI_SSGBLUP	2168.95 (116.30)	–	3.75 × 10^–3^ (5.95 × 10^–4^)	571.73 (56.91)	422.21 (61.32)	5092.61 (93.86)	1.54 × 10^–2^ (5.69 × 10^–3^)	0.26 (0.04)	0.19 (0.04)	–
BI_ SSGBLUP	2174.28 (110.43)	− 1.28 (0.31)	3.58 × 10^–3^ (5.26 × 10^–4^)	568.85 (54.42)	418.41 (70.61)	5080.37 (100.25)	1.56 × 10^–2^ (4.22 × 10^–3^)	0.26 (0.03)	0.19 (0.04)	− 0.46 (0.13)
UNI_MF-SSGBLUP	2305.03 (122.34)	–	3.79 × 10^–3^ (6.31 × 10^–4^)	570.11 (56.87)	415.72 (61.25)	5077.82 (93.91)	1.56 × 10^–2^ (5.72 × 10^–4^)	0.27 (0.03)	0.20 (0.03)	–
BI_MF-SSGBLUP	2311.95 (118.92)	− 1.37 (0.22)	3.86 × 10^–3^ (5.91 × 10^–4^)	567.00 (60.40)	411.53 (57.75)	5065.37 (88.41)	1.55 × 10^–2^ (5.01 × 10^–4^)	0.28 (0.03)	0.20 (0.03)	− 0.46 (0.12)

Variance components for genetic parameters correspond to the usual genetic variance (in MF-SSGBLUP, all the variance components are multiplied by ($1 - \gamma_{Y} )/2$), which is the variance among unrelated individuals in the base population

$\sigma_{a\_ADG}^{2}$ is the additive genetic variance for ADG, $\sigma_{a\_FCR}^{2}$ is the additive genetic variance for FCR; $\sigma_{{a\_ADG,{ }a\_FCR}}$ is genetic covariance between ADG and FCR; $\sigma_{litter}^{2}$ is the variance of litter effect for ADG; $\sigma_{e\_ADG}^{2}$ is the residual variance for ADG; $\sigma_{e\_FCR}^{2}$ is the residual variance for FCR

Numbers between brackets are the standard errors of the corresponding parameters

For the LL population, estimates of the additive genetic variance for ADG and FCR were similar for the four methods (Tables 2 and 3). Estimates of heritabilities for ADG ($h_{a\_ADG}^{2}$) and FCR ($h_{a\_FCR}^{2}$) were also almost identical for the four methods, with $h_{a\_ADG}^{2}$ ranging from 0.23 to 0.24 and $h_{a\_FCR}^{2}$ ranging from 0.10 to 0.11, but with very high standard errors (SE), i.e. ranging from 0.04 to 0.05 for $h_{a\_ADG}^{2}$ and from 0.05 to 0.07 for $h_{a\_FCR}^{2}$. The estimate of the genetic correlation between ADG and FCR was higher negative with *BI_MF-SSGBLUP* ($r_{g}$ = − 0.27, SE = 0.10) than with *BI_ SSGBLUP* ($r_{g}$ = − 0.19, SE = 0.09).

Results for the YY population were similar to those for the LL population. Variance component estimates were similar for the four methods, except for estimates of the additive genetic variance for ADG and FCR, which were slightly greater with the use of metafounders (*UNI_MF-SSGBLUP* and *BI_MF-SSGBLUP*) than for the two SSGBLUP methods. Estimates of heritability were consistent for the four methods and ranged from 0.26 to 0.28 for ADG, with SE from 0.03 to 0.04, and from 0.19 to 0.20 for FCR, with SE from 0.03 to 0.04. The *BI_MF-SSGBLUP* and *BI_ SSGBLUP* methods resulted in the same estimate of the *g*enetic correlation between ADG and FCR (− 0.46, with SE ranging from 0.12 to 0.13).

### Predictive ability

**Fig. 1:**
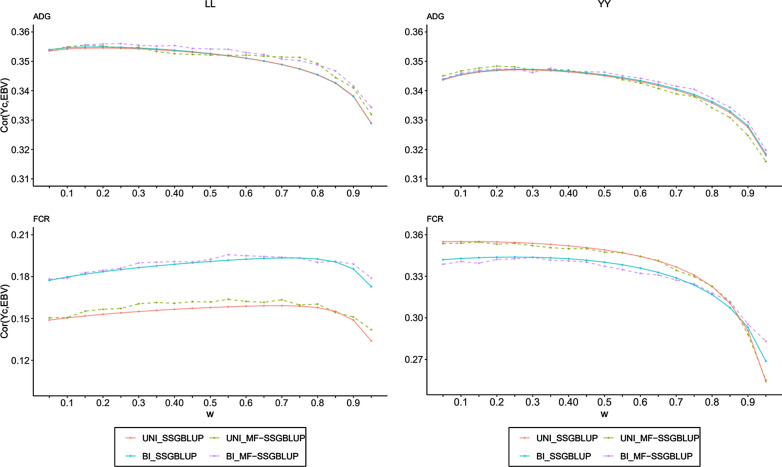
Accuracy for ADG and FCR. The effect of the weighting factor (ω) on accuracy for ADG and FCR for the four methods for two populations, where the X-axis represents ω ranging from 0.05 to 0.95, and the Y-axis indicates the correlation coefficients between corrected phenotypes (${\text{Y}}_{c}$) and genomic estimated breeding values ($\hat{a}$)

The effect of the weighting factor (ω) on accuracy for ADG and FCR for the four methods are shown in Fig. 1. For the LL population, the four methods resulted in nearly the same accuracy for ADG, with the highest accuracy equal to 0.355. The optimal weighting factors (ω) were 0.20, 0.15, 0.20, and 0.25 for *UNI_SSGBLUP*, *UNI_MF-SSGBLUP*, *BI_SSGBLUP*, and *BI_MF-SSGBLUP*, respectively. For FCR, the predictive ability was slightly higher for the bivariate (0.19) than for the univariate methods (0.16). The optimal ω was 0.70 for *UNI_SSGBLUP* and *BI_SSGBLUP*, and 0.55 for *UNI_MF-SSGBLUP* and *BI_MF-SSGBLUP*. For the YY population, the curves of the predictive ability for ADG as a function of ω for the four methods nearly overlapped at the top (Fig. 1). The highest predictive ability (0.347) was obtained when ω was set to 0.30 for all four methods. For FCR, the univariate models performed slightly better than the bivariate models when ω was lower than 0.85, i.e. the predictive ability was highest, 0.355, with ω equal to 0.10 and 0.15 for *UNI_SSGBLUP* and *UNI_MF-SSGBLUP*, respectively, and 0.344 with a ω of 0.25 and 0.30 for *BI_SSGBLUP* and *BI_MF-SSGBLUP*, respectively.

Taken together, when the same value of ω was used, the predictive ability did not significantly differ between the four methods. The predictive ability of method *BI_MF-SSGBLUP* was slightly superior compared to that of the other methods.

In order to compare the predictive abilities for genotyped and non-genotyped animals, the LL and YY validation populations were divided into a genotyped and a non-genotyped subgroup, and the predictive abilities for ADG and FCR were calculated at the optimal ω for each subgroup. The results are in Table 4. As shown in Fig. 1, the optimal ω for SSGBLUP and MF-SSGBLUP was nearly the same for a given trait and population. Thus, we only investigated the predictive abilities for the genotyped and non-genotyped subgroups at the optimal ω of 0.20, 0.70, 0.25, and 0.10 for to ADG in LL, FCR in LL, ADG in YY, and FCR in YY, respectively. Based on Table 4, for both the univariate and the bivariate models, the predictive abilities of SSGBLUP and MF-SSGBLUP were similar for both subgroups but the predictive ability was higher for the genotyped subgroups than for the non-genotyped subgroups.

**Table 4:** Accuracies of genomic predictions for average daily gain (ADG) and feed conversion ratio (FCR) in different validation groups for univariate (UNI) or bivariate (BI) single-step genomic prediction models with or without metafounders (MF), each with their optimal weighting factor (ω) in the two populations

Population	Trait	Validation group	*UNI_SSGBLUP*	*UNI_MF-SSGBLUP*	*BI_SSGBLUP*	*BI_MF-SSGBLUP*
Landrace	ADG (ω = 0.20)	All	0.355^a^	0.356^a^	0.355^a^	0.356^a^
		Genotyped	0.410^a^	0.410^a^	0.410^a^	0.410^a^
		Non-genotyped	0.327^a^	0.327^a^	0.326^a^	0.327^a^
	FCR (ω = 0.70)	All	0.192^a^	0.193^a^	0.193^a^	0.196^b^
		Genotyped	0.200^a^	0.206^b^	0.201^a^	0.206^b^
		Non-genotyped	0.167^a^	0.170^a^	0.179^b^	0.180^b^
Yorkshire	ADG (ω = 0.25)	All	0.347^a^	0.347^a^	0.347^a^	0.347^a^
		Genotyped	0.395^a^	0.395^a^	0.394^a^	0.394^a^
		Non-genotyped	0.309^a^	0.309^a^	0.309^a^	0.308^a^
	FCR (ω = 0.10)	All	0.355^a^	0.355^a^	0.344^b^	0.344^b^
		Genotyped	0.370^a^	0.367^a^	0.357^b^	0.356^b^
		Non-genotyped	0.118^a^	0.121^a^	0.117^a^	0.114^a^

The four methods are *UNI_SSGBLUP*, *UNI_MF-SSGBLUP*, *BI_SSGBLUP* and *BI_MF-SSGBLUP*

Validation groups are the validation population including all the young pigs born between 1 May 2016 and 1 February 2017, and two subgroups of genotyped and non-genotyped animals

The results for ADG and FCR bivariate single-step genomic prediction models are obtained with their optimal ω respectively

Superscript letters indicate significant differences (p < 0.05) by the Hotelling–Williams t-test

**Fig. 2:**
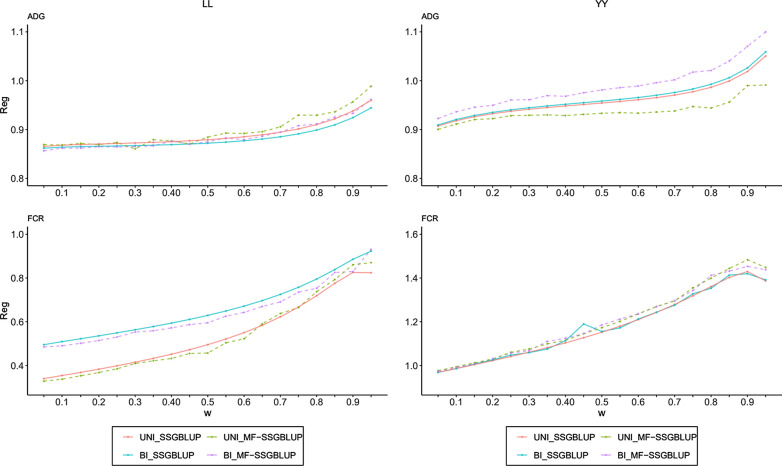
Bias for ADG and FCR. The effect of the weighting factor (ω) on bias of genomic predictions for ADG and FCR for the four methods for two populations, where the X-axis represents ω ranging from 0.05 to 0.95, and the Y-axis indicates the regression coefficients of corrected phenotypes (${\text{Y}}_{c}$) on genomic estimated breeding values ($\hat{a}$)

The effect of the weighting factor (ω) on bias of genomic predictions for ADG and FCR for the four methods are shown in Fig. 2. In general, the regression coefficient increased as ω increased. For the LL population, when ω was set to 0.95, the regression coefficients were closest to 1. For ADG, the effect of ω on the regression coefficient was nearly the same for the four methods; for FCR, the regression coefficients deviated more from 1 for the univariate models than for the bivariate models. For the YY population, the regression coefficient for ADG with method *BI_MF-SSGBLUP* was closest to 1 with a ω of 0.70, indicating that this method was the best among the four methods in terms of bias. For FCR, there were no dramatic differences in bias between the four methods. When ω was set to 0.15, the bias of genomic predictions was lowest for all four methods. When the optimal ω was used, the bias of genomic predictions was lower for the non-genotyped subgroup than for the genotyped subgroup for all four methods (see Table 5).

**Table 5:** Biases of genomic predictions for average daily gain (ADG) and feed conversion ratio (FCR) in different validation groups for univariate (UNI) or bivariate (BI) single-step genomic prediction models with or without metafounders (MF), each with their optimal weighting factor (ω) in the two populations

Population	Trait	Validation group	*UNI_SSGBLUP*	*UNI_MF-SSGBLUP*	*BI_SSGBLUP*	*BI_MF-SSGBLUP*
Landrace	ADG (ω = 0.20)	All	0.960	0.989	0.945	0.962
		Genotyped	0.733	0.739	0.732	0.738
		Non-genotyped	0.838	0.839	0.829	0.828
	FCR (ω = 0.70)	All	0.824	0.871	0.923	0.931
		Genotyped	0.542	0.479	0.565	0.524
		Non-genotyped	1.078	1.283	0.764	0.881
Yorkshire	ADG (ω = 0.25)	All	0.999	0.991	1.006	1.002
		Genotyped	0.743	0.742	0.750	0.760
		Non-genotyped	0.867	0.866	0.870	0.886
	FCR (ω = 0.10)	All	1.004	1.012	1.006	1.008
		Genotyped	0.472	0.488	0.483	0.502
		Non-genotyped	1.006	0.996	1.005	0.999

The four methods are *UNI_SSGBLUP*, *UNI_MF-SSGBLUP*, *BI_SSGBLUP* and *BI_MF-SSGBLUP*

Validation groups are the validation population including all the young pigs born between 1 May 2016 and 1 February 2017, and two subgroups of genotyped and non-genotyped animals

The results for ADG and FCR for bivariate single-step genomic prediction models are obtained with their optimal ω respectively

## Discussion

In this work, we compared predictive abilities of genomic predictions for two economically important production traits, ADG and FCR, in DanBred Landrace and Yorkshire populations between using regular SSGBLUP and MF-SSGBLUP. First, we estimated ${\upgamma }$ to construct the ${\mathbf{H}}\left( {\upgamma } \right)^{ - 1}$ matrix for MF-SSGBLUP. Then, we compared genetic parameters estimates and predictive abilities between SSGBLUP and MF-SSGBLUP, both for univariate and bivariate models. This study also explored the optimal weighting factor, ω, on pedigree relationships for ADG and FCR for both SSGBLUP and MF-SSGBLUP.

### Estimation of γ for MF-SSGBLUP

For use in MF-SSGBLUP, we obtained estimates of γ of 0.605 and 0.553 for the LL and YY populations, respectively, which differed from those reported previously for the same populations (0.756 for LL and 0.730 for YY) [12]. Legarra et al. [5] have pointed out that several factors can affect estimates of γ, such as effective population size and the SNP panel used. The populations used in the current study and in previous studies [12] originated from the same ancestors, thus the effective population size was not responsible for the difference in γ estimates. However, in our study we used fewer SNPs (about 37,500 SNPs) than in the previous study (41,000 SNPs), which is due to a difference in the quality control criteria on minor allele frequencies between the two studies, which was 0.05 for both the LL and YY populations, while Xiang et al.’s [12] used a threshold of 0.01. Thus, the additional SNPs in their study had a lower allele frequency, which increased the percentage of homozygotes and resulted in greater estimates of γ [12].

### Genetic parameters

When using MF-SSGBLUP and SSGBLUP, although both the univariate and bivariate models produced similar genetic (co)variance estimates, the slightly smaller standard errors for the bivariate models indicated that bivariate models can reduce the uncertainty of genetic parameter estimates compared to the univariate models. Estimates of the genetic correlation between ADG and FCR were based on the bivariate models. Although MF-SSGBLUP resulted in a higher negative (− 0.27) estimate than SSGBLUP (− 0.19) for the LL population, this difference was not significant. And MF-SSGBLUP and SSGBLUP resulted in the same estimate for the YY population.

Estimates of heritability were similar for the four methods within a population, and the heritability estimate for FCR was considerably lower for the LL (about 0.11) than the YY (about 0.20) population. In the literature, a range of heritabilities for FCR has been reported. For example, Kavlak and Uimari [29] reported an estimate of 0.28 for Finnish Yorkshires; Dube et al. [30] estimates ranging from 0.21 to 0.27 for South African Yorkshires; and Hoque et al. [31] reported an estimate of 0.27 for Duroc pigs. In this study, the SE of the estimate of heritability for FCR was substantial (about 0.05). Thus, further studies are needed to compute the relevant genetic parameters based on larger datasets.

### Predictive ability

Martini et al. [32] have reported that a high weighting factor ω increases the variance of EBV. Therefore, we explored the optimization of ω. However, predictive ability was only slightly changed by ω. The ω with the highest predictive ability was defined as the optimal ω. For ADG, the optimal ω was almost the same (around 0.2) for the LL and YY populations, but for FCR, the optimal ω was substantially higher for the LL (around 0.7) than for the YY (around 0.1) population. This may be caused by a difference in the proportions of genetic variances accounted for by 50 K SNPs between the two populations. Miao et al. [33] showed that the genes that affect FCR differ between LL and YY [33], thus the gene-linked SNPs may capture different proportions of the total genetic variance for the two populations.

In this study, predictive ability was higher than that reported in a previous study [9], likely because of the larger population size both in terms of number of phenotypic records and amount of genomic information. In our study, the estimated accuracy for ADG and FCR with SSGBLUP was higher than that reported for total number of piglets born in a previous study on the same populations [26], likely because the latter trait has a lower heritability than ADG and FCR. As shown in Table 4, the predictive ability for ADG and FCR was higher for the genotyped subgroups than for the non-genotyped subgroups for all four methods, consistent with previous reports [9, 12]. However, Guo et al. [34] reported that the predictive reliability of non-genotyped subgroups was higher than that of genotyped subgroups for total number of piglets born and litter size at day 5 after birth in the LL population, which they attributed to pre-selection of the genotyped animals, which reduced the correlations between phenotypes and genotypes. In this study, although genotyped animals were also pre-selected, the number of genotyped animals after pre-selection was still large enough to provide sufficient information to make genomic predictions of genotyped animals more accurate than those of non-genotyped animals. Furthermore, the regression coefficients were lower for the genotyped subgroups than for the non-genotyped subgroups, while the bias of non-genotyped subgroups was similar to that of the whole validation population.

Theoretically, genomic relationships should be constructed using base allele frequencies [4] and some studies [35] have used estimated base allele frequencies to construct the genomic relationship matrix for genomic prediction. Thus, predictive abilities and bias were also evaluated for a bivariate SSGBLUP model with a genomic relationship matrix constructed using estimated base allele frequencies, using the optimal ω of 0.20, 0.70, 0.25, and 0.10 for ADG in LL, FCR in LL, ADG in YY, and FCR in YY, respectively. We obtained the following predictive abilities and bias: 0.245 and 0.743, respectively for ADG in LL pigs, 0.114 and 0.642 for FCR in LL pigs, 0.198 and 0.567 for ADG in YY pigs, and 0.141 and 0.550 for FCR in YY pigs. These values were much lower than those obtained with SSGBLUP with a genomic relationship matrix constructed using observed allele frequencies. There are two possible explanations why the use of a genomic relationship matrix constructed using base allele frequencies resulted in worse predictive performance than the methods evaluated in this study: first, we used estimates of variance components using SSGBLUP with a genomic relationship matrix that was constructed using the observed allele frequencies; second, the estimated base allele frequencies may not have been accurate. However, we found that the performance of MF-SSGBLUP was at least as good as that of SSGBLUP, which indicates that the variance of the estimated base allele frequencies was reliable, since the self-relationship γ was estimated using this variance.

Overall, MF-SSGBLUP performed slightly better for genomic prediction for ADG and FCR than SSGBLUP, although the differences were not always statistically significant. This finding is in agreement with the theory [5]. Thus, overall we recommend the use of MF-SSGBLUP for genomic evaluation in pigs.

## Conclusions

The single-step genomic evaluation method with a metafounder (MF-SSGBLUP) was successfully implemented for genomic prediction of ADG and FCR in Danish Landrace and Yorkshire populations and was found to be slightly superior to the regular single-step method. The optimal weighting factor (ω) on pedigree information was slightly different for SSGBLUP versus MF-SSGBLUP. Based on our results, the bivariate model with a metafounder approach is recommended to estimate breeding values for correlated production traits such as ADG and FCR.

## Data Availability

The datasets in the current study are not publicly available since they are owned by SEGES, but are available from the corresponding author upon reasonable request and if agreed by SEGES.
